# Dehydroepiandrosterone Attenuates Astroglial Activation, Neuronal Loss and Dendritic Degeneration in Iron-Induced Post-Traumatic Epilepsy

**DOI:** 10.3390/brainsci13040563

**Published:** 2023-03-27

**Authors:** Chandra Prakash, Shyam Sunder Rabidas, Jyoti Tyagi, Deepak Sharma

**Affiliations:** 1 Neurobiology Laboratory, School of Life Sciences, Jawaharlal Nehru University, New Delhi 110067, India; 2 Nanobiotechnology Laboratory, School of Biotechnology, Jawaharlal Nehru University, New Delhi 110067, India

**Keywords:** epilepsy, seizures, dehydroepiandrosterone, neuronal loss, astroglial activation, dendritic degeneration

## Abstract

Iron-induced experimental epilepsy in rodents reproduces features of post-traumatic epilepsy (PTE) in humans. The neural network of the brain seems to be highly affected during the course of epileptogenesis and determines the occurrence of sudden and recurrent seizures. The aim of the current study was to evaluate astroglial and neuronal response as well as dendritic arborization, and the spine density of pyramidal neurons in the cortex and hippocampus of epileptic rats. We also evaluated the effect of exogenous administration of a neuroactive steroid, dehydroepiandrosterone (DHEA), in epileptic rats. To induce epilepsy, male Wistar rats were given an intracortical injection of 100 mM solution (5 µL) of iron chloride (FeCl_3_). After 20 days, DHEA was administered intraperitoneally for 21 consecutive days. Results showed epileptic seizures and hippocampal Mossy Fibers (MFs) sprouting in epileptic rats, while DHEA treatment significantly reduced the MFs’ sprouting. Astroglial activation and neuronal loss were subdued in rats that received DHEA compared to epileptic rats. Dendritic arborization and spine density of pyramidal neurons was diminished in epileptic rats, while DHEA treatment partially restored their normal morphology in the cortex and hippocampus regions of the brain. Overall, these findings suggest that DHEA’s antiepileptic effects may contribute to alleviating astroglial activation and neuronal loss along with enhancing dendritic arborization and spine density in PTE.

## 1. Introduction

Epilepsy is a common neurological disorder affecting about 65 million people worldwide [1]. The major characteristics of this neurological disorder are the onset of sudden and recurrent seizures resulting from changes in neuronal hyperexcitability and electrical discharge [2]. It has been estimated that among the total number of acquired epilepsy cases, 20% are of post-traumatic epilepsy (PTE) that occurs after a brain trauma [3]. The studies on human patients and animal models demonstrated that epileptogenesis involves a plethora of cellular and molecular changes, viz., alteration of brain circuitry, changes in the levels of neurotransmitters and receptors, aberrant gene expression, neurogenesis, and neuronal loss [4,5]. The mechanism of epileptogenesis in PTE is complex. It seems to be linked with multiple pathophysiological changes that can alter the balance between neuronal excitation–inhibition, making the brain more susceptible to producing recurrent and spontaneous seizures [6].

Seizures induced by intracortical injection of iron chloride (FeCl_3_) in rodents reproduce characteristics of PTE in humans, resulting from severe head trauma [7]. This model has been characterized regarding electrophysiological, neurobehavioral, morphological, biochemical, and molecular aspects. The model has also been extensively used to investigate novel therapeutic strategies for PTE [8,9,10,11,12].

Compelling evidence suggests that Mossy Fibers (MFs) sprouting, inflammation, astroglial activation, and neuronal degeneration are interesting disease-modifying targets of epilepsy [13,14]. The activation of glial cells is recognized to have a critical role in the onset and progression of epilepsy [15,16,17]. The activation and proliferation of astrocytes and microglia produce inflammatory cytokines that can influence neuronal hyperexcitability and degeneration [18,19]. Besides this, cognitive and behavioral impairments in epilepsy are the consequences of disruption of the neuronal network [20]. Thus, their attenuation can provide a strong rationale for investigating novel therapeutic agents with long-term anti-seizure potential.

Dendritic arbors and spines are important morphological entities of excitatory synaptic neurons, and changes in these entities are implicated in various neurological disorders. Particularly, any changes in dendritic arbors and spines have been reported to directly affect epileptogenesis and seizure onset [21]. Emerging evidence from animal models and human specimen suggest that dendritic pathologies such as abnormal arbors and loss of spines are reported in multiple forms of epilepsy [22,23,24]. Moreover, investigations conducted on patients with seizures, such as cardinal symptom, in pathological conditions like hippocampal sclerosis, tumors, and microdysgenesis, have also reported dendritic degeneration and spines loss [25,26]. However, the causes or consequences of PTE, especially in FeCl_3_-induced experimental epilepsy, have not been determined till now.

Most currently available anti-seizure medications (ASMs) provide only symptomatic suppression of seizures without modifying the pathophysiology of epilepsy. The long-term use of these ASMs can also exert several adverse effects [27]. Hence, drugs with neuroprotective and disease-modifying properties are of particular interest since they may exert antiepileptic effects, reduce disease severity, and attenuate associated comorbidities. Research focusing on the therapeutic modulation of physiological and molecular changes can provide novel strategies with an effect beyond seizure suppression [28]. The development of efficient alternative therapeutics that might lessen the harmful changes occurring throughout the epileptogenic process is, therefore, urgently needed [29].

Dehydroepiandrosterone (DHEA) is an important circulating steroid hormone, synthesized novo by the adrenals, gonads, and brain. This steroid interacts with various receptors of neural growth factors and consequently exerts neuroprotective effects [30]. Evidence suggests that DHEA benefits various neurological disorders, including epilepsy [31]. Our earlier research revealed that DHEA’s antiepileptic potential is accompanied by its antioxidative, antiapoptotic, and voltage-gated ion channels [8,9] modulatory properties. In addition, DHEA has been proven to diminish inflammation caused by microglia in mouse models of neuroinflammation and in vitro cultures of microglia [32], suggesting its anti-inflammatory potential. Given the above-mentioned characteristics of DHEA antiepileptic and disease-modifying effect of exogenous DHEA, treatment for 21 days was investigated by evaluating astroglial activation, neuronal loss, and dendritic morphology in the cortex and hippocampus of a rat model of experimental PTE.

## 2. Materials and Methods

### 2.1. Animals

Male Wistar rats (weighing 220–250 g) used for the experimentation were procured from Central Laboratory Animal Resources, Jawaharlal Nehru University, New Delhi, India. Throughout the experiment, the animals were housed in a pathogen-free standard environment (22 ± 2 °C) with a 12-h light/dark cycle at 50–60% humidity and had free access to food and water. All experiments were approved by the Institutional Animal Ethics Committee (IAEC) of Jawaharlal Nehru University, New Delhi. Every effort was made to minimize the number of rats, their pain, and suffering.

### 2.2. Epilepsy Model

The experimental model of PTE was developed by administering FeCl_3_ intracortically to rats as per the procedure described in previous reports [8,9]. Stereotaxic surgery was performed under an aseptic condition in a stereotaxic apparatus (Stoelting, Wood Dale, IL, USA) using 4% volatile isoflurane (Baxter, Deerfield, MA, USA) as an anesthetic. An incision was made along the scalp midline, and peri-cranial muscles and fascia were gently removed to expose the skull. Then, a burr hole of 0.5 mm diameter was drilled for FeCl_3_ injection (AP-1, ML-1, DV-1.5 mm). A total of 5 μL of 100 mM FeCl_3_ solution (prepared in normal saline) was injected with the help of an injector cannula over 5 min (at a rate of 1 μL/min) via a microsyringe (Hamilton Company, Reno, NV, USA) fixed in a micro syringe pump controller (Stoelting, Wood Dale, IL, USA). Post injection, the cannula was ejected, and the burr hole was sealed with sterile bone wax. In the cortical area, four stainless steel surface electrodes were stereotaxically implanted (AP + 2 and −2, ML + 2 and −2, DV −1.5 mm from bregma). Moreover, at the CA1 area of the hippocampus, one intra-cerebral bipolar wire electrode was placed (AP-2.8, ML-2.5, DV-2.71 mm from bregma). Finally, the incision was stitched and Nebasulf^®^ sprinkling powder (Pfizer Ltd., Bangalore, Karnataka, India) was applied to the surgical site for a few days to avoid infection.

### 2.3. Experimental Design

A total of 18 rats were randomly assigned to three experimental groups. (1) Control: (n = 6) rats received an intracortical saline injection as FeCl_3_ solvent. (2) Epileptic: (n = 6) rats received an intracortical injection of FeCl_3_ solution as described above. (3) Epileptic + DHEA: (n = 6) rats received an intracortical injection of FeCl_3_ solution and DHEA treatment for 21 consecutive days.

**Figure 1:**
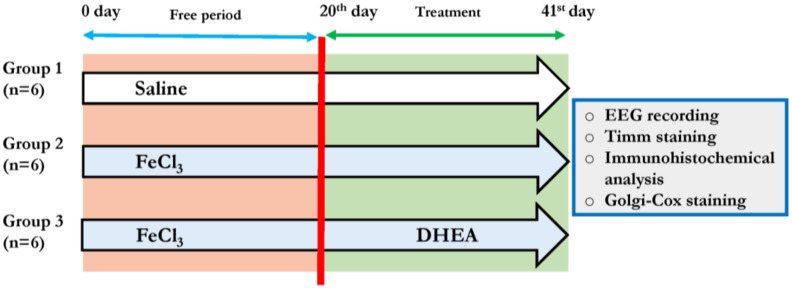
Schematic illustration of the treatment paradigm and assay parameters of the study.

After 20 days of FeCl_3_ injection, DHEA, solubilized in 0.1% dimethylsulphoxide (Sigma Aldrich, St. Louis, MO, USA), was injected intraperitoneally (30 mg/kg b. wt.) for the next 21 days (Figure 1). DHEA dose and duration used in this study are consistent with our laboratory’s earlier publications indicating its antiepileptic effect at 7, 14, and 21 days of treatment [8,33]. As 21 days of DHEA administration showed a stronger antiepileptic effect, the same was chosen.

### 2.4. EEG Recordings and Analysis

Electroencephalography (EEG) video recordings were used to measure epileptiform seizures in experimental rats. All rats were accustomed to the recording equipment and chamber for three days prior to the recordings beginning. The recordings were taken during the light phase, and animals were given at least 5 min to settle down. The EEG signals were filtered through 1 Hz to 100 Hz bandpass, amplified by an amplifier (P511 AC preamplifiers), and recorded using PolyVIEW 16 Data Acquisition System (Grass Technologies, West Warwick, RI, USA).

### 2.5. Tissue Preparation for Histopathology and Immunofluorescence Analysis

At the end of the treatment period, rats (n = 3) were anesthetized with ketamine: a xylazine mixture (100 mg/kg: 10 mg/kg) and transcardially perfused with saline (0.9% NaCl) and 2% paraformaldehyde (PFA). Intact brains were dissected out and post-fixed in 2% PFA overnight. Then, dehydrated by passing through 10, 20, and 30% of sucrose solutions. Coronal sections (10 μm thickness) of the brain were cut through the dorsal part of the hippocampus in a cryostat (Leica CM 1860, Leica Biosystems, Nussloch GmbH. Heidelberger Str., Germany) and collected on gelatin-coated slides for further analysis.

### 2.6. Timm Staining

Tissue sections were air dried, followed by incubation in Timm’s working solution, consisting of 50% Arabic gum (120 mL), 2 M citrate buffer (20 mL), 0.5 M hydroquinone (60 mL), and 17% silver nitrate (1 mL) for 60 min in the dark. Following washes, sections underwent graded alcohol dehydration and xylene clearing. After that, sections were cover-slipped with DPX mounting media (Fisher Scientific, Mumbai, India), left to dry overnight, and photographed using a light microscope (Motic Instruments Co. Ltd., Chengdu, China). Timm staining intensity was quantified using densitometric analysis in the hippocampus (DG region) of the brain using FIJI software (Imagej 1.53t, NIH, Bethesda, MD, USA; http://fiji.sc/fiji accessed on 5 January 2022). The area and white background were used to normalize the grayscale staining’s mean density. Then, using a linear scale, density ratings were nonparametrically graded.

### 2.7. Immunofluorescence Staining

The brain sections were washed in PBS and then treated with 5% Triton-X100 for 10 min. The non-specific antigens were then blocked with 3% normal goat serum (Abcam, Cambridge, UK). Subsequently, sections were covered with primary antibodies, such as mouse anti-GFAP (1:100, Invitrogen, Carlsbad, CA, USA) and rabbit anti-NeuN (1:100, Cell Signaling Technology, Danvers, MA, USA), and placed at 4 °C overnight. After three PBS washes, sections were incubated at room temperature with Alexa Fluor 488-conjugated goat anti-mouse (1:200, Invitrogen, Carlsbad, CA, USA) and Alexa Fluor 594-conjugated goat anti-rabbit (1:200, Invitrogen, Carlsbad, CA, USA secondary antibodies. Sections were washed again with PBS, and the nucleus was stained with 4′,6-diamidino-2-phenylindole (DAPI) (Sigma, St. Louis, MO, USA). Next, sections were covered with Fluoromount^TM^ Aqueous Mounting Media (Sigma, St. Louis, MO, USA). Finally, images were taken under a fluorescent microscope (Nikon Eclipse 90iT, Tokyo, Japan).

### 2.8. Golgi-Cox Staining

Rats (n = 3) were perfused as described above. Isolated brains were rinsed with PBS and separated into two equal hemispheres. The staining was done according to the previously described procedure of Zhong et al. [34]. Briefly, 10 mL of impregnation solution was poured over the brain tissue and incubated for two days. Then, the solution was exchanged and incubated for the next two weeks. After impregnation, the excess solution was removed and wiped with tissue paper. Then, the tissue was transferred to 15 mL of chilled cryoprotectant solution with gentle shaking and stored at 4 °C. Next, the cryoprotectant solution was replaced after 24 h and again stored at 4 °C until the tissue sank into the bottom. After that, coronal sections measuring 80 µm thickness were cut using a cryostat and collected on slides coated with gelatin. Sections were then washed twice in distilled water for two min each, followed by a 10 min of soak in 20% ammonia solution in darkness. Then, sections were washed with distilled water, treated for 5 min with 1% sodium thiosulfate, and washed with distilled water again. Subsequently, sections were cleared with xylene after being dehydrated with graded alcohol solutions (50, 75, 95, and 100%). Finally, sections were cover-slipped with the aid of DPX and allowed to dry. Individual pyramidal neurons were imaged using a light microscope (Motic Instruments Co. Ltd., Chengdu, China) at 40× for arborization analysis and 60× for dendritic spines.

### 2.9. Analysis of Dendritic Morphology and Spine Density

A total of 10 pyramidal neurons from both cortex and hippocampus of each animal (n = 3) were captured under 40× objective and traced by the Simple Neurite Tracer (SNT) feature of Fiji software (https://imagej.net/plugins/simple-neurite-tracer/ accesed on 5 January 2022). The concentric ring method of Sholl [35] was used to analyze the branching pattern and complexity of dendrites. The number of dendritic intersections at each concentric ring from the center of the soma up to 200 µm of radial distance was used to calculate the complexity of neurons.

A total of 20 primary and secondary branches from the apical dendrites of five randomly chosen pyramidal neurons were photographed using a 60× objective lens to calculate spine density. Dendritic spines were counted using Fiji software. Primary and secondary dendritic branches chosen were 2 to 3 µm thick, at least 10 µm long, and focused on a single plane. Spines with two heads were counted as two spines. Two researchers unaware of group identities conducted individual counts, and the average results are represented as the number of spines/10 µm of dendritic length [36].

### 2.10. Statistical Analysis

Data were analyzed using one-way Analysis of Variance (ANOVA) and two-way ANOVA (for Sholl analysis) with Holm-Sidak post hoc test. Statistical analyses were conducted using SigmaStat 3.5 software (Systat Software Inc., San Jose, CA, USA). A probability value ≤ 0.05 was considered statistically significant.

## 3. Results

### 3.1. DHEA Treatment Alleviates Epileptiform Seizures in Epileptic Rats

**Figure 2:**
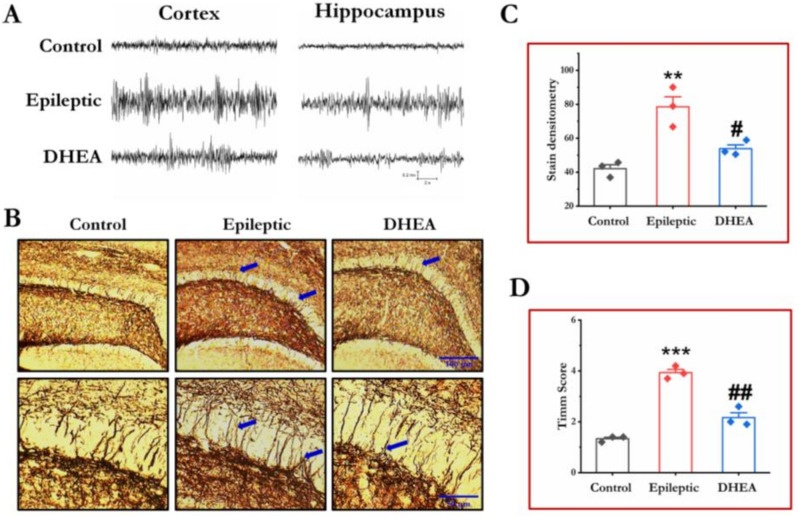
Representative EEG samples of 20 s duration from the cortex and hippocampus of control, epileptic, and DHEA-treated rats (**A**). Timm’s stating images showing Mossy Fibers (indicated by arrows) in the dentate gyrus (DG) region of rats (**B**). Quantitative data analysis indicates lower stain density (**C**) and Timm’s score (**D**) in DHEA-treated rats with respect to epileptic rats. ** *p* ≤ 0.01, *** *p* ≤ 0.001, significantly different from controls; # *p* ≤ 0.05, ## *p* ≤ 0.01, significantly different from epileptic rats. ANOVA F values for stain densitometry: 17.659 and Timm’s score: 24.035.

EEG recordings from each group were examined to measure epileptiform seizure activity. EEG recordings from epileptic rats showed increased seizure activity in their cortex and hippocampus. As illustrated in Figure 2A, epileptic seizures can be easily distinguished in 20 s EEG stretches because of their distinct single spikes, polyspikes, and spike waves. DHEA treatment for 21 days significantly reduced spike waves, single spikes, and polyspikes associated with epileptiform activity (Figure 2A).

### 3.2. DHEA Treatment Reduces MFs Sprouting in Epileptic Rats

Timm’s staining was used to determine MFs sprouting in the DG region of the hippocampus. Stain intensity and Timm’s score were calculated to assess the severity of MFs sprouting. We discovered that control rats showed no evidence of MFs sprouting, whereas epileptic rats had a clear band of Timm staining in the DG’s molecular layer. DHEA-treated rats had significantly lower MFs sprouting than epileptic rats. Densitometric analysis of axonal sprouting and Timm’s score confirmed that DHEA treatment could have significantly reduced the severity of MFs sprouting (Figure 2B–D).

### 3.3. DHEA Treatment Attenuates Activation of Astrocytes

**Figure 3:**
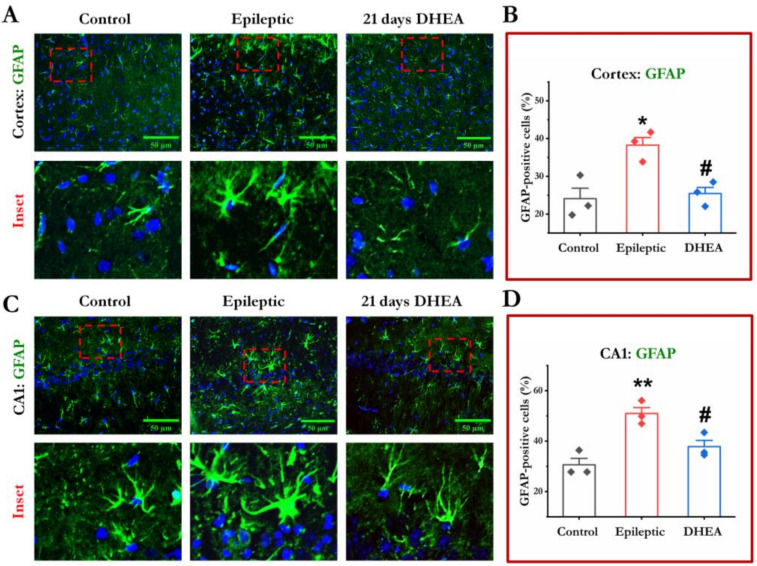
Immunofluorescence analysis of reactive astrocytes in the cortex and hippocampus regions of control, epileptic and DHEA-treated rats. Representative photomicrographs showing GFAP-positive cells (green) (**A**,**C**). Percentage of GFAP-positive cells in the cortex (**B**) and hippocampus (**D**) of experimental rats. Data are expressed as mean ± SD (n = 3 in each group). * *p* ≤ 0.05, ** *p* ≤ 0.01, significantly different from control group; # *p* ≤ 0.05, significantly different from epileptic group. ANOVA F values for percentage of GFAP-positive cells: cortex 8.356; hippocampus 13.558.

GFAP is a well-known marker of reactive astrocytes, and we investigated the effect of DHEA on astroglial activation by evaluating its immunoreactivity in the experimental rats. Control rats showed a lower percentage of GFAP-positive cells in the cortex and hippocampus, and the cells resembled resting astroglia. In contrast, epileptic rats showed a higher percentage of GFAP-positive cells, morphologically similar to reactive astrocytes. The percentage of GFAP-positive cells was dramatically decreased in epileptic rats treated with DHEA. These findings imply a considerable diminution of activated astrocytes in the epileptic rats treated with DHEA (Figure 3).

### 3.4. DHEA Treatment Protects Loss of Neurons

**Figure 4:**
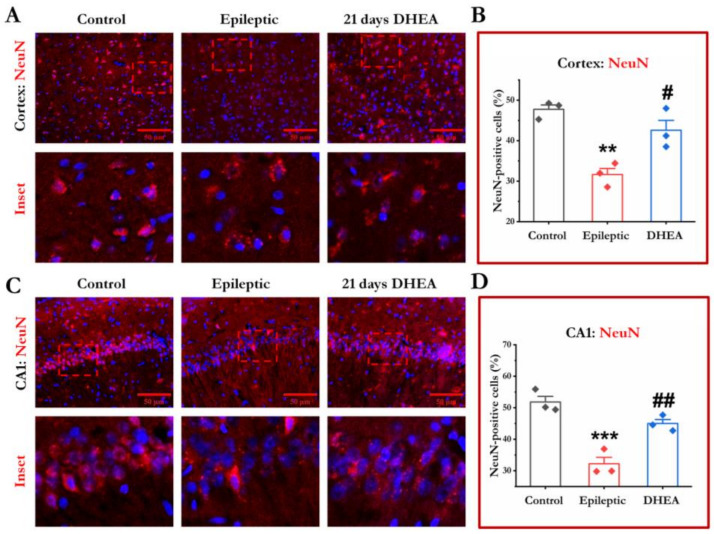
Immunofluorescence analysis of neuronal loss in the cortex and hippocampus regions of control, epileptic and DHEA-treated rats. Representative photomicrographs showing NeuN-positive cells (red) (**A**,**C**). Percentage of NeuN-positive cells in the cortex (**B**) and hippocampus (**D**) of experimental rats. Data are expressed as mean ± SD (n = 3 in each group). ** *p* ≤ 0.01, *** *p* ≤ 0.001 significantly different from control group; # *p* ≤ 0.05, ## *p* ≤ 0.01 significantly different from epileptic group. ANOVA F values for percentage of NeuN-positive cells: cortex 16.623; hippocampus 25.107.

To assess DHEA’s neuroprotective effect, NeuN immunoreactivity was measured in the cortex and hippocampus of experimental rats. Control rats showed a higher percentage of NeuN-positive cells in the cortex and the hippocampus. The epileptic rats showed a significant decrease in NeuN-positive neurons in both regions of the brain. Interestingly, compared to epileptic animals, DHEA injection dramatically increased the percentage of NeuN-positive cells. There was no significant difference in any brain regions between the control and DHEA-treated rats (Figure 4).

### 3.5. DHEA Treatment Rescues Dendritic Arborization

**Figure 5:**
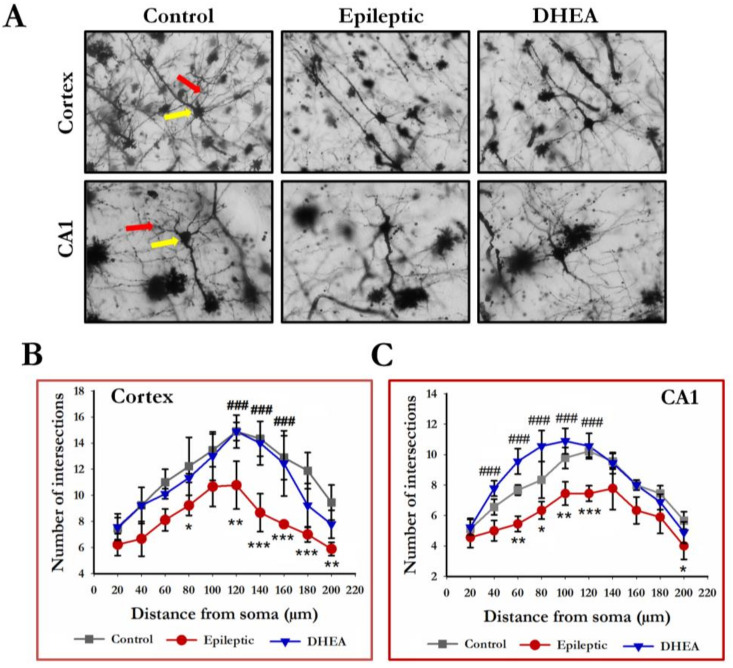
Dendritic arborization in the cortex and hippocampus of control, epileptic and DHEA-treated rats. Representative Golgi-Cox-stained neurons photomicrographs from the cortex and hippocampus captured under 40× magnification showing dendrites (red arrows) and soma (yellow arrows) (**A**). Quantification of dendritic arborization by evaluating the total number of intersections at each concentric ring away from the soma in the cortex (**B**) and hippocampus (**C**) of experimental rats. Data are expressed as mean ± SD (n = 3 in each group). * *p* ≤ 0.05, ** *p* ≤ 0.01, *** *p* ≤ 0.001 significantly different from control group; ### *p* ≤ 0.001 significantly different from epileptic group. ANOVA F values for the number of intersections: cortex 47.897; hippocampus 49.751.

Dendritic arborization of pyramidal neurons seemed to be affected during the course of epileptogenesis. Morphological analysis of cortical and hippocampal CA1 pyramidal neurons in control rats showed long and extensively arborized dendrites. We discovered a considerable reduction in dendritic arbors, as well as disorientation of apical and basal dendrites of pyramidal neurons in epileptic rats. In contrast, DHEA treatment in epileptic rats significantly elevated dendritic arborization in both brain regions, suggesting the positive impact of DHEA in preventing impaired dendritic arborization in PTE (Figure 5).

### 3.6. DHEA Restores Dendritic Spines

**Figure 6:**
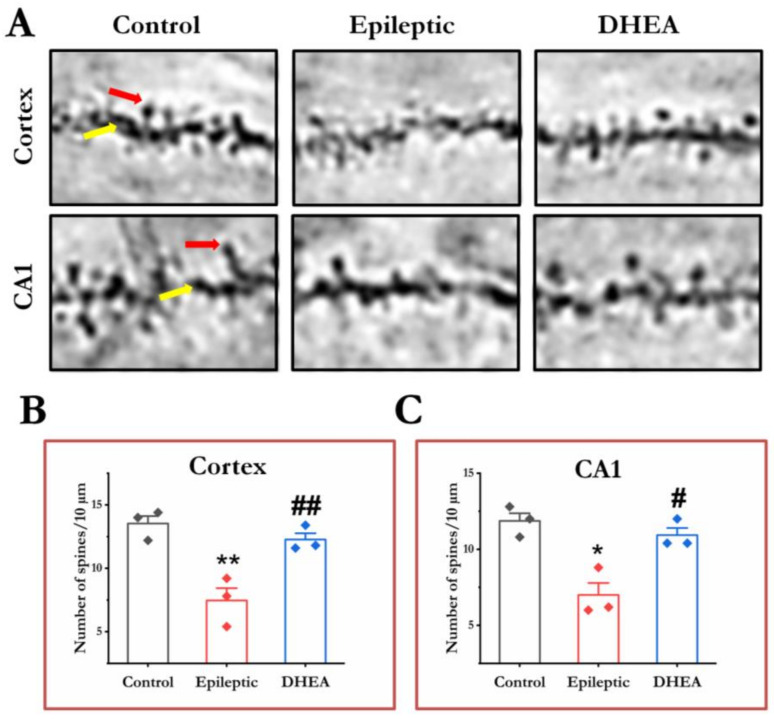
Morphological analysis of dendritic spines in the cortex and hippocampus of control, epileptic and DHEA-treated rats. Representative photomicrographs of Golgi-Cox-stained neurons from the cortex and hippocampus captured under 60× magnification showing apical dendrites (yellow arrows) and spines (red arrows) (**A**). Average number of dendritic spines in pyramidal neurons of the cortex (**B**) and hippocampus (**C**) of experimental rats. Data are expressed as mean ± SD (n = 3 in each group). * *p* ≤ 0.05, ** *p* ≤ 0.01, significantly different from control group; # *p* ≤ 0.05, ## *p* ≤ 0.01 significantly different from epileptic group. ANOVA F values for several dendritic spines: cortex 15.260; hippocampus 12.070.

Spine density analysis in apical dendrites of cortical and hippocampal pyramidal neurons showed a major population of mushroom-shaped spines in control rats. Further, the results revealed a considerably lower number of dendritic spines in epileptic rats both in the cortex and hippocampus regions. On the other hand, epileptic rats treated with DHEA showed a considerably higher number of dendritic spines in the apical branches of cortical and hippocampal pyramidal neurons (Figure 6).

## 4. Discussion

Available evidence from clinical and pre-clinical studies indicates that epileptogenesis is a multifactorial process. In the case of PTE, seizures occur following head trauma. The possible pathophysiological mechanisms of PTE have been investigated in rodent models produced by intracortical FeCl_3_ injection [7]. Several biochemical and molecular changes seem to be involved in the development of epilepsy after FeCl_3_ injection, where cellular oxidative damage, apoptosis [8,37], levels of neurotransmitters and receptors [33], and voltage-gated ion channels [9,10,12] have major roles in the exacerbated excitability of neurons. Previous studies from our laboratory investigated the antiepileptic effects of DHEA along with its antioxidative and neuromodulatory role in PTE [8,9,33]. However, its effect on astroglial activation and dendritic degeneration remains unexplored.

The findings from this study indicate that the negative effects of FeCl_3_-induced experimental PTE were reversed by exogenous treatment with DHEA. The results demonstrated that epileptic episodes in FeCl_3_-injected rats were significantly reduced after 21 days of DHEA administration. DHEA treatment decreased the frequency and amplitude of high-amplitude of EEG events, which may have improved epileptiform seizures. The MFs are axons of dentate granule cells that, under normal conditions, create synapses with pyramidal cells of the hippocampus. In the epileptic brain, these axons lose their connecting targets, innervate the inner molecular layer of DG, and cause epileptogenesis via the formation of recurrent excitatory circuits [38]. Researches on both humans and animals suggest that the sprouting of MFs is one of the most common neuropathological features of epilepsy. In the present study, we discovered MFs’ sprouting in the DG of rats with FeCl_3_ injection. These consequences were ameliorated by DHEA treatment for 21 days, showing its antiepileptic potential, and are consistent with other research [8,9,33,37]. Suggesting the antiepileptic effect of DHEA, as evidenced by a considerable decrease of epileptic episodes in electrophysiological investigations.

The mechanism of epileptogenesis seems to be associated with the activation of glial cells, evident from the increased expression of astrocytic and microglial markers in experimental epilepsy [16,39]. According to a study by Lee et al. [40], astrogliosis is linked with glutamate overexpression in hippocampal sclerosis, which raises neuronal excitability and causes seizures. Moreover, the reactivation of astrocytes results in the rapid depletion of GABAergic neurons’ synapses and cases of hyperexcitability of the hippocampal circuitry [41]. Hence, the possible mechanism by which reactive glial cells promote epileptogenesis seems modulated by neuronal excitability and inflammation [19]. A growing body of evidence from earlier studies provides insight into the importance of astroglial activation in epilepsy [19,42]. The current investigation reported significantly higher number of GFAP-positive cells in the cortex and hippocampus of epileptic rats. These results are consistent with previous reports [16,39] and indicate activation of astrocytes in both regions of the brain. Next, we evaluated the effectiveness of DHEA on astroglial activation and found that DHEA effectively reduced astrogliosis. This evidence supports the notion that neuroactive compounds, such as DHEA, can attenuate the process of astroglial activation in an experimental model of PTE.

PTE has also been reported to exhibit degeneration and death of cortical and hippocampal neurons [8]. Some studies reported that developing and matured neurons degenerate in patients and animal models of experimental epilepsy [43,44]. In this study, we observed that FeCl_3_-induced epilepsy is linked with significant neuronal loss in the cortex and hippocampus regions, as evident from the lower number of NeuN-positive cells in both regions. These findings agree with previous studies that show the extent of neuronal loss in different epilepsy models [8,45,46]. DHEA has been reported to act as a neurotropic or neuroprotective factor under different pathological conditions, including epilepsy [31]. Indeed, we found that DHEA administration results in an increased number of NeuN-positive neurons in both regions of the brain, which suggests the neuroprotective effect of DHEA in PTE. These findings are consistent with our prior report, indicating the neuroprotective effect of DHEA in FeCl_3_-induced epilepsy through rescuing degenerative neurons and reducing apoptotic cell death [8].

The arborization of dendrites defines their connectivity and is pivotal for integrating information and synaptic plasticity. Numerous human and animal studies demonstrated structural abnormalities in dendrites that could contribute to neuronal dysfunction, cognitive and behavioral deficits, and epileptogenesis [21,47,48]. The degeneration of dendrites has been recognized as a common feature of epileptic tissues in both humans and animals [21,49]. The stabilization of dendritic structure could become a future therapeutic strategy for epilepsy. We examined the morphological changes of neurons using Golgi-Cox staining and found fewer arborized neurons in the cortex and hippocampus of epileptic rats. A line of studies performed on multiple forms of epilepsy reported abnormal morphological changes, viz. reduced dendrite arborization and length [14,22,24]. Interestingly, exogenous treatment of DHEA alleviated dendritic retraction caused in epileptic rats. Further, we observed that DHEA treatment rescued dendritic arborization, although some dendritic retraction persisted. Similarly, DHEA has also shown improved neuronal plasticity and facilitation of dendritic development in middle-aged rats exposed to chronic mild stress [50]. Overall, these results indicate that the antiepileptic potential of DHEA may also be accompanied by improved dendritic arbors leading to increased surface area and connectivity of dendrites.

The structural remodeling of synapses can be analyzed by counting the dendritic spines. Previously, various reports demonstrated significantly decreased dendritic spines in the pyramidal neurons of epilepsy patients and animal models [14,21,22,23,24]. These findings strongly suggest that abnormalities in dendritic spines can also play an important role in the pathophysiology of epilepsy. We also observed significantly decreased spine density in the cortex and hippocampus of epileptic rats, which is consistent with other results and suggests a correlation between dendritic spine abnormalities and seizures in PTE. However, DHEA treatment reduced the density of dendritic spines in both regions of the brain, thus, providing evidence that DHEA’s antiepileptic effects may also be influenced by the recovery of dendritic spine density.

## 5. Conclusions

In conclusion, our results exhibit that DHEA possesses definite and substantial neuroprotective effects on the epileptic brain. The treatment of DHEA may have decreased the sprouting of MFs in the hippocampus, suggestive of a reduced incidence of seizures in PTE. Further, the steroid may have escorted an anti-seizure effect by ameliorating astroglial activation, neuronal loss, and dendritic degeneration in the cortex and hippocampus regions of the brain in PTE.

## Data Availability

All the relevant data generated and analyzed during the current study are available upon reasonable request.
